# Oral health status among newly arrived refugees in Germany: a cross-sectional study

**DOI:** 10.1186/s12903-018-0600-9

**Published:** 2018-08-03

**Authors:** Monzer Solyman, Andrea-Maria Schmidt-Westhausen

**Affiliations:** Department of Oral Medicine, Dental Radiology and Oral Surgery, Charite Universitatsmedizin Berlin, Assmannshauser Str. 4-6, 14197 Berlin, Germany

**Keywords:** Oral health, Refugees, Decayed, missing and filled teeth index, Knowledge, attitude and practice survey

## Abstract

**Background:**

The objectives of this study were to determine the status of oral health among newly arrived refugees in Germany and to explore their knowledge, attitude and practices on oral hygiene.

**Methods:**

All participants (*n* = 386) were adults, 18–60 years of age, coming from Syria and Iraq and registered as refugees in Germany within one year prior to the enrollment in the study. Clinical oral assessments in addition to a survey on knowledge, attitude and practice were carried out. The survey was conducted through a questionnaire translated into Arabic.

**Results:**

Eighty seven point 5 % of the participants had untreated caries. The mean DMFT score was 6.38 with DT, MT and FT showing mean scores of 4.00, 1.46 and 0.92 respectively. Seventy nine percent had bacterial plaque in all six sextants, 60 % had calculus in at least three sextants and 6 % showed various magnitudes of enamel fluorosis. DMFT score was significantly associated with age (Regression Coefficient 0.031, *P*-value < 0.001) and with education (Regression Coefficient − 0.019, *P*-value 0.037) and females had significantly less missing teeth (Regression Coefficient-0.398, *P*-value 0.001) compared to males. The participants had in general high levels of knowledge and attitude on oral hygiene. The findings however showed a gap between their knowledge and practice.

**Conclusions:**

The findings show high prevalence of untreated caries and poor oral hygiene among newly arrived refugees in Germany. The study recommends to lay emphasis on motivation in oral health promotion campaigns among refugees and to provide them with adequate guidance, preferably in their native language, on how to access oral health care in the host country.

## Background

Since the beginning of the internal conflicts in Syria and Iraq, the humanitarian crisis along with the collapse of the health systems in these countries have led to a dramatic increase in the influx of refugees and asylum-seekers into the European Union [1]. Of those, Germany has received the highest number of asylum applications among the member states of the European Union according to the United Nations High Commissioner for Refugees [2].

Oral conditions including dental caries and periodontal diseases have a significant impact on the quality of life of the individuals and a high economic burden on the health system in the hosting country [3]. Accordingly, many studies were carried out within the last two decades to investigate the oral health of refugee populations including Somali refugees in the USA [4], African and Eastern European refugees in the USA [5], Vietnamese refugees in Australia [6], Liberian refugees in Ghana [7] and immigrants and refugees in Italy [8]. These studies have shown a high prevalence of dental caries, periodontal diseases and poor oral hygiene. Furthermore, the status of oral health appeared to considerably differ among different refugee populations depending on the geographical region of origin [6, 8]. The studies suggested pre-arrival conditions to be equally important as those during the arrival and post-arrival period in determining the state of oral health of refugees.

This study is among the first to be specifically conducted on the oral health of refugees coming from Syria and Iraq in Germany and Europe. The particularity of the study population, including the existence of a functioning health and educational system in the country of origin prior to the war and the behavioral and dietary cultural norms, mainly high sugar intake [9], high prevalence of tobacco smoking [10] and low alcohol consumption [11], may suggest particular needs. Therefore, the objective of this study was to determine the status of oral health among newly arrived refugees coming from Syria and Iraq in Germany and to explore their knowledge, attitude and practice regarding oral hygiene.

## Methods

A cross-sectional study was conducted between July and December 2016, in which clinical oral assessments of 386 newly arrived refugees from Syria and Iraq were carried out. Participants were recruited in refugee reception centers (174 participants), shelters (189 participants) and private practices (23 participants) in Berlin. The recruitments took place on site at the same day of the clinical examinations. Upon the informed consent of the participants, clinical oral examinations were carried out. These were directly followed by a structured interview conducted in their native language to identify the knowledge, attitude and practices (KAP) on oral hygiene. Participants were adults, 18–60 years of age, and registered as refugees in Germany within one year prior to the enrollment in the study. Children, adolescents and elderly were excluded.

### Sample size calculation

The sample size was determined using the following formula for cross-sectional studies [12]:

$$ \mathrm{n}={\mathrm{Z}}^2\ \mathrm{P}\ \left(1-\mathrm{P}\right)/{\mathrm{d}}^2 $$

where n = sample size, Z = 1.96 (level of confidence 95%), *P* = 0.5 (expected proportion in population) and d = 0.05 (precision).

The sample size (*n* = 386) represented a proportion of 0.3% of the total number (*n* = 115,647) of Syrians and Iraqis who had their application for asylum accepted in Germany in 2015 [13].

### Clinical oral assessments

The clinical examinations were carried out in artificial lighting with two mouth mirrors. All clinical examinations were done by one examiner MS (an Arabic native speaking dentist with a dental license in Germany). Participants were not explicitly asked to brush their teeth prior to the examination. The clinical assessment form for adults was initially developed by WHO and was modified accordingly for the study [14]. In this regard, invasive diagnostic procedures including periodontal probing were excluded, as several studies have suggested that patients with periodontitis may be at high risk of bacteremia following periodontal probing without preceding antibiotic prophylaxis [15–17].

Decayed, Missing and Filled Teeth Index (DMFT) was used to explore caries prevalence. Bacterial plaque and calculus were recorded as present or not present, for which the labial and lingual surfaces of 6 index teeth (16 or 17, 11, 26 or 27, 36 or 37, 31 and 46 or 47) were examined. Cases with missing index teeth were excluded and the recordings were then transferred into two scales, one for plaque and another for calculus, each has 7 ordinal values, where 0 = not present and 6 = present in all 6 sextants.

Dental trauma was recorded upon presence, for which six categories were accordingly registered, where 0 = No sign of injury, 1 = Treated injury, 2 = Enamel fracture only, 3 = Enamel and dentine fracture, 4 = Pulp involvement and 5 = Missing tooth due to trauma [18].

Enamel fluorosis was recorded using Dean’s index, for which six categories were accordingly registered, where 0 = Normal, 1 = Questionable “*slight aberrations from the translucency of normal enamel*”, 2 = Very mild “*small, opaque, paper-white areas scattered irregularly over the tooth but involving less than 25% of the labial tooth surface*”, 3 = Mild “*more extensive but covers less than 50% of the tooth surface*”, 4 = Moderate “*marked wear and brown stain*” and 5 = Severe “*enamel surfaces are badly affected*” [18].

The intervention urgency was classified based on the clinical findings into five categories, where 0 = No treatment needed, 1 = Preventive or routine treatment needed, 2 = Prompt treatment (including scaling) needed, 3 = Immediate (urgent) treatment needed due to pain or infection of dental and/or oral origin and 4 = Referred for comprehensive evaluation or medical/dental treatment (systemic condition) [18].

The intra-rater reliability was examined in a convenience sample, for which twenty three participants, all of them recruited in private practices, were examined on two different occasions. The two examinations were carried out by the same examiner conducting the study. Both examinations were done in the standard study settings. The two examinations were then compared showing an intra-class correlation coefficient of 0.94.

### KAP survey on oral hygiene

The participants were interviewed immediately after the clinical assessments. The interviews were carried out in their native language (Arabic) following a questionnaire exploring their knowledge, attitude and practices on oral hygiene. Similar questionnaires were used and developed through previous studies on oral hygiene in children and adolescents [19–22]. However, the questionnaire used in our study was proposed by WHO [23] for adults. The questionnaire was further adjusted for the purposes of this study and included eleven questions, among which:

Three question were on participants´ knowledge concerning tooth brushing and flossing:

- What should one use for cleaning his/her teeth?
- When should one brush his/her teeth?
- In addition to brushing, should one floss his/her teeth?

Four questions were on their attitude towards oral health:

- Do you think that brushing your teeth improves your dental health?
- Do you think dental problems can affect general health?
- How often should one visit a dentist?
- How would you describe the state of your teeth and gums?

Four questions were on their practices respecting oral hygiene:

- What do you use for cleaning your teeth?
- How do you brush your teeth?
- How often do you clean your teeth?
- Do you use toothpaste containing fluoride?

Participants´ answers were then scored, in which they were given + 1 for each correct answer, − 1 for each incorrect answer and 0 for giving no answer. This resulted in three scores (discrete variables accepting negative values), one for knowledge (ranging from − 3 to + 3), one for attitude (ranging from − 4 to + 4) and one for practice (ranging from − 4 to + 4).

With respect to their perception of own oral health, participants were asked to rate the state of their teeth and gums on a scale of 5 degrees, where 1 = Poor, 2 = Average, 3 = Good, 4 = Very good and 5 = Excellent. A comparison was then made between the self-perceived assessment and a clinical assessment made by the examiner on the same scale. Taking into consider the subjectivity involved in the examiner’s clinical assessment and the participant’s interpretation of the wording in the scale, the participant’s answer was only considered incorrect (overestimation or underestimation) when it differed from the examiner’s evaluation by two degrees or more.

### Statistical analysis

The primary data set was analyzed using Stata 13.0 software (Stata, Texas, USA). DMFT scores were presented in means and standard deviations while other indices were presented in percentages. Negative binomial regression model NBRM was used to test for the association of count outcomes including DMFT, DT, MT and FT with the socio-demographic characteristics of the study population including age, country of origin, gender and education. Upon the comparison of mean observed and predicted count of our DMFT data using the Akaike’s information criterion AIC and Bayesian information criterion BIC, the test showed a preference of NBRM with a very strong evidence over other models usually used for count outcomes.

In order to preserve the information contained in the ordering, ordered logistic regression OLR was used to test for the association of ordinal outcomes including calculus and plaque with socio-demographic characteristics. OLR may have cells that are too small for reliable estimates, nevertheless, two tests were applied to examine the goodness of fit of the model: an ordinal version of the Hosmer-Lemeshow test (*P*-value = 0.944 for calculus and P-value = 0.899 for plaque showing a good fit for both) and the Lipsitz test (P-value = 0.991 for calculus and P-value = 0.368 for plaque showing a good fit for both). In addition, a second model for ordinal outcomes, Multilevel mixed-effects generalized linear model (Family: ordinal, Link: complementary log-log), was added in order to enhance the validity of the analysis. The two models have shown matching significant associations for all characteristics (Table 4).

For discrete variables accepting negative values (KAP scores), multivariate linear regression was used to test for the association with socio-demographic characteristics of the study population.

A *P*-value of < 0.05 was considered significant.

## Results

### Socio-demographic characteristics of the study participants

Most of the participants were young males with 80.1% males and 42% between 18 and 24 years of age (Table 1). About 60 % were Syrians compared to 38.1% coming from Iraq. Concerning education, Only 5.4% had no education, 18.4% had primary education (1–6 years in school), 28.2% had preparatory education (7–9 years in school), 25.1% had secondary education (10–12 years in school) had 22.8% had at least one year of university.

**Table 1:** Socio-demographic characteristics of the study participants (*n* = 386)

Characteristics		Number (Percentage)
Country of origin	Syria	239 (61.9)
	Iraq	147 (38.1)
Gender	Male	309 (80.1)
	Female	77 (19.9)
Age group in years	18–24	162 (42.0)
	25–29	66 (17.1)
	30–34	55 (14.2)
	35–44	69 (17.9)
	more than 44	34 (8.8)
Educational level (years in school)	No education	21 (5.4)
	Primary school (1–6)	71 (18.4)
	Preparatory school (7–9)	109 (28.2)
	Secondary school (10–12)	97 (25.1)
	University (more than 12)	88 (22.8)

### Oral health indices and KAP survey

The mean DMFT score was 6.38 with DT, MT and FT showing mean scores of 4.00, 1.46 and 0.92 respectively. In this context, only two participants were fully edentulous. The presence of bacterial plaque and calculus was abundant with almost 80 % of the participants having plaque in all six sextants and almost 60 % having calculus in at least three sextants. Regarding the dental trauma, the vast majority of the participants (95.6%) had no sign of injury. Six percent of the participants showed various magnitudes of enamel fluorosis, among which only two (0.5%) were at severe stage. No participant had a systemic condition that required referral for comprehensive medical evaluation. Nonetheless, more than half needed prompt treatment and about one tenth had either pain or infection that required immediate treatment. With regard to KAP survey, most participants had good knowledge on tooth brushing. On the contrary, only one fifth answered that the additional use of dental floss is necessary. With regard to their attitude, less than one third believed in the relationship between oral and general health and less than half believed they should have regular checkups by a dentist. Almost two thirds had a proper perception of their own oral health compared to one third giving rather over or underestimations. The participants´ practices on oral hygiene were generally weak with more than half using the wrong method to brush their teeth and the majority cleaning their teeth less than twice a day. Detailed results on oral health indices and KAP survey are described in Table 2.

**Table 2:** Oral health status and KAP survey (n = 386)

Oral health index		Number
Caries prevalence, mean (SD)	DMFT	6.38 (5.058)
	DT	4.00 (3.352)
	MT	1.46 (3.388)
	FT	0.92 (1.694)
Presence of bacterial plaque, n (%)	No plaque observed	2 (0.55)
	Plaque observed on one sextant	20 (5.49)
	Plaque observed on two sextants	7 (1.92)
	Plaque observed on three sextants	41 (11.26)
	Plaque observed on four sextants	1 (0.27)
	Plaque observed on five sextants	6 (1.65)
	Plaque observed on six sextants	287 (78.85)
Presence of calculus, n (%)	No calculus observed	35 (9.59)
	Calculus observed on one sextant	107 (29.32)
	Calculus observed on two sextants	4 (1.10)
	Calculus observed on three sextants	107 (29.32)
	Calculus observed on four sextants	1 (0.27)
	Calculus observed on five sextants	2 (0.55)
	Calculus observed on six sextants	109 (29.86)
Dental trauma, n (%)	No sign of injury	369 (95.60)
	Treated injury	4 (1.04)
	Enamel fracture only	7 (1.81)
	Enamel and dentine fracture	5 (1.30)
	Pulp involvement	0 (0.0)
	Missing tooth due to trauma	1 (0.26)
Enamel fluorosis, n (%)	Normal	363 (94.04)
	Questionable	4 (1.04)
	Very mild	3 (0.78)
	Mild	9 (2.33)
	Moderate	5 (1.30)
	Severe	2 (0.52)
Intervention urgency, n (%)	No treatment needed	18 (4.66)
	Preventive or routine treatment	124 (32.12)
	Prompt treatment (including scaling)	202 (52.33)
	Immediate treatment due to pain or infection	42 (10.88)
	Referred for comprehensive evaluation	0 (0.0)
KAP Questionnaire		
Question	Answer	Number
What should one use for cleaning his/her teeth? n (%)	Toothbrush a	379 (98.19)
	Miswak b	7 (1.81)
	Others (finger, charcoal or wooden toothpicks) b	0.0 (0.0)
When should one brush his/her teeth? n (%)	Once or less a day b	74 (19.17)
	Twice or more a day a	312 (80.83)
In addition to brushing, should one floss his/her teeth? n (%)	Yes a	76 (19.69)
	No b	267 (69.17)
	I don’t know	43 (11.14)
Do you think that brushing your teeth improves your dental health? n (%)	Yes a	352 (91.19)
	No b	26 (6.74)
	I don’t know	8 (2.07)
Do you think dental problems can affect general health? n (%)	Yes a	249 (64.51)
	No b	115 (29.79)
	I don’t know	22 (5.70)
How often should one visit a dentist? n (%)	Regularly a	175 (45.34)
	Whenever there is a problem b	207 (53.63)
	I don’t know	4 (1.04)
How would you describe the state of your teeth and gums? n (%)	Participant’s answer showed a proper perception of his/her own oral health a	239 (61.92)
	Participant’s answer showed a considerable over or underestimation of his/her own oral health b	141 (36.5)
	Participant answered: I don’t know	6 (1.55)
What do you use for cleaning your teeth? n (%)	Toothbrusha	382 (99.22)
	Miswak b	3 (0.78)
	Others (finger, charcoal or wooden toothpicks) b	0.0 (0.0)
How do you brush your teeth? n (%)	Up and down circular motion, involving gums a	176 (45.60)
	Left to right, horizontal direction b	204 (52.85)
	I don’t know	6 (1.55)
How often do you clean your teeth? n (%)	Once or less a day b	158 (40.93)
	Twice or more a day a	228 (59.07)
Do you use toothpaste containing fluoride? n (%)	Yes a	11 (2.85)
	No b	8 (2.07)
	I don’t know	367 (95.08)

*DMFT* Decayed Missing or Filled Teeth, *DT* Decayed Teeth, *MT* Missing Teeth, *FT* Filled Teeth, *SD* Standard Deviation, (%) All percentages in this table are valid percentages (cases with missing index teeth were excluded)

^a^ Correct answer

^b^ Incorrect answer

### Association between main oral health indices and socio-demographic characteristics

Age was found to have a significant association with DMFT score (*P*-value < 0.001) and its components DT, MT and FT (*P*-values 0.047, < 0.001 and < 0.001 respectively). Older participants had higher DMFT scores compared to their younger counterparts. On the contrary, there was no significant association of the country of origin or of gender with DMFT scores (*P*-value 0.873 and 0.975 respectively) although females had significantly less missing teeth (*P*-value 0.001) compared to males. With regard to education, a significant association was found with DMFT score (RC -0.019, *P*-value 0.037). The more years in school participants had, the less decayed (RC -0.021, *P*-value 0.047), the less missing (RC -0.069, *P*-value 0.003) and the more filled teeth (RC 0.050, *P*-value 0.045) they showed (Table 3). Concerning oral hygiene, the presence of bacterial plaque was found to be significantly less with younger age (OLR *P*-value 0.009) and higher education (OLR *P*-value 0.028). It was also found to be significantly less among females (OLR *P*-value 0.013) compared to males. On the other hand, there was a significant increase in calculus presence with age (OLR *P*-value < 0.001). Females showed significantly lower levels of calculus (OLR *P*-value < 0.001) compared to males, as well as Iraqis (OLR *P*-value 0.018) compared to Syrians. In addition, those with higher education had less calculus (OLR *P*-value 0.019) compared to those with lower education (Table 4).

**Table 3:** Association of DMFT and its components with socio-demographic characteristics (Negative binomial regression model, *n* = 386)

Variables	DMFT	DT	MT	FT
	Regression Coefficient (Standard error), *P*-value			
Increase in age per year (continuous variable)	0.031 (0.003), < 0.001	0.008 (0.004), 0.047	0.089 (0.010), < 0.001	0.043 (0.013), < 0.001
Country of origin (ref. Syrians)	−0.013 (0.078), 0.873	− 0.165 (0.091), 0.069	0.657 (0.203), 0.112	0.001 (0.222), 0.997
Gender (ref. Male)	0.003 (0.091), 0.975	0 .070 (0.106), 0.507	−0.398 (0.250), 0.001	0.382 (0.246), 0.121
Increase in education per year (continuous variable)	−0.019 (0.009), 0.037	−0.021 (0.010), 0.047	− 0.069 (0.023), 0.003	0.050 (0.025), 0.045
Overall model Pseudo R-squared value, P-value	0.035, < 0.001	0.006, 0.022	0.093, < 0.001	0.012, 0.001

*DMFT* Decayed Missing or Filled Teeth, *DT* Decayed Teeth, *MT* Missing Teeth, *FT* Filled Teeth

**Table 4:** Association of calculus and plaque with socio-demographic characteristics (*n* = 365^a^)

Variables	Model 1 (OLR)		Model 2 (Multilevel mixed-effects GLM)	
	Calculus	Plaque	Calculus	Plaque
	Odds ratios (95% Confidence Interval), *P*-value		Regression Coefficient (Standard error), P-value	
Increase in age per year ^b^	1.060 (1.035–1.085), < 0.001	1.046 (1.011–1.082), 0.009	0.028 (0.006), < 0.001	0.018 (0.007), 0.008
Country of origin (ref. Syrians) ^b^	0.610 (0.410–0.917), 0.018	0.856 (0.496–1.477), 0.576	− 0.350 (0.126), 0.006	−0.053 (0.123), 0.667
Gender (ref. Male) ^b^	0.298 (0.182–0.487), < 0.001	0.471 (0.260–0.852), 0.013	−0.658 (0.151), < 0.001	− 0.332 (0.144), 0.021
Increase in education per year ^b^	0.946 (0.904–0.991), 0.019	0.930 (0.872–0.992), 0.028	−0.031 (0.013), 0.018	− 0.032 (0.013), 0.019
Pseudo R-squared value, P-value	0.048, < 0.001	0.029, 0.002		
Wald Chi-squared, P-value			42.02, < 0.001	15.70, 0.003
Hosmer-Lemeshow test: Estimate, P-value ^c^	37.729, 0.944^d^	40.382, 0.899^d^		
Lipsitz test: Estimate, P-value ^c^	2.022, 0.991^d^	9.787, 0.368^d^		

*OLR* Ordered logistic regression, *GLM* Generalized linear model

^a^ Cases with missing index teeth were excluded

^b^ The two models are showing matching significant associations

^c^ Goodness of fit test for ordinal logistic regression model

^d^ P-value is showing the model to be a good fit

### Association between KAP scores and socio-demographic characteristics

No significant association was found of age, neither with knowledge nor with practice. However, the older the participants were, the higher scores on attitude they showed (*P*-value 0.005). Iraqis showed significantly lower scores on attitude compared to their Syrian counterparts (*P*-value 0.005). Yet, the differences in knowledge and practice were insignificant. Females had significantly higher scores on knowledge (*P*-value 0.034) and practice (*P*-value 0.001), nonetheless, no significant difference between males and females on attitude was found. Education was significantly associated with KAP scores; the higher education the participants had, the higher the level of knowledge, attitude and practice (*P*-value < 0.001) they presented (Table 5).

**Table 5:** Association of knowledge, attitude and practice with socio-demographic characteristics (Multivariate linear regression) (n = 386)

Variables	Knowledge	Attitude	Practice
	Regression Coefficient (Standard error), P-value		
Increase in age per year (continuous variable)	− 0.002 (0.006), 0.729	0.029 (0.010), 0.005	−0.010 (0.008), 0.187
Country of origin (ref. Syrians)	−0.157 (0.124), 0.206	−0.595 (0.213), 0.005	− 0.132 (0.160), 0.408
Gender (ref. Male)	0.312 (0.146), 0.034	0.093 (0.250), 0.710	0.633 (0.189), 0.001
Increase in education per year (continuous variable)	0.055 (0 .0140), < 0.001	0.0920 (0.024), < 0.001	0.086 (0.018), < 0.001
Overall model R-squared value, P-value	0.064, < 0.001	0.089, < 0.001	0.096, < 0.001

## Discussion

In accordance with previous studies on refugees in other western countries [5, 6, 8, 24, 25], the findings have shown high prevalence of dental caries and poor oral hygiene among newly arrived refugees in Germany.

Seventy nine percent of the participants had untreated caries while 78.8% showed bacterial plaque in all 6 sextants and 60% showed calculus in at least three sextants. However, the participants have surprisingly presented a lower DMFT mean score (6.99) in comparison with the mean national index (DMFT 11.2) for the comparable age group (35–44 years) in Germany [26]. Nonetheless, the magnitude of DMFT score among Germans owed its height to a rather high number of filled teeth (FT 8.6 compared to 1.10 in refugees) while it owed its height among refugees to a rather high number of untreated caries (DT 3.81 compared to 0.5 in Germans). Both had an almost equal number of missing teeth (MT 2.07 in refugees to 2.1 in Germans).

The high prevalence of untreated caries among refugees may be attributed to pre-arrival conditions, as well as to limited access to oral health care after arrival in the host country. Studies have suggested various factors contributing to the limited access, among which, not being able to afford treatment, lacking orientation within the new health system, being socially isolated, facing language barriers and a general low emphasis on oral health and promotion during the resettlement period [6, 24, 25, 27–29]. According to the German law [30], refugees in Germany have full access to dental care as German citizens after the official acknowledgment of their asylum status. During the period preceding the acceptance of their application for asylum, they are only eligible for primary care for acute conditions or pain [31].

Within the resettlement period, the priority of refugees clearly lies on the reestablishment in the host country. This would most likely lead to consequences on their oral health behavior including the low utilization of dental services [6, 8]. Therefore, it is important for decision-makers in the host country to target this population at risk as early as possible. This could be achieved through providing access to immediate oral assessment and treatment upon arrival. Likewise, it is important to establish an active inclusion of refugees in the existing health structures.

Available data on fluorosis have suggested a prevalence of about 7.1 to 11.3% among German adolescents. In comparison, our study showed a prevalence of 6% among adult refugees coming from Syria and Iraq. Fluorosis among Germans was mainly attributed to the early start of tooth brushing [32], On contrary, Fluorosis among refugees is probably due to a high-level exposure to fluoride in drinking water back in the country of origin as Syria and Iraq belong to the known fluoride belts according to WHO [33].

In general, there were no significant differences between Syrians and Iraqis on oral health indices including the prevalence of caries and bacterial plaque. Iraqis however had significantly less calculus accumulations. Although Iraq was for a longer period of time affected by war in comparison with Syria, yet most of the Iraqi refugees in Germany came from the northern part of the country. This part was until recent years relatively stable and the health system there showed similar indices to that of Syria before the eruption of the current civil conflict [34]. On the other hand, the findings have demonstrated that other socio-demographic factors including age, gender and education were statistically relevant to the status of oral health among refugees. Older and less educated participants presented more caries and poorer oral hygiene. Females had significantly less missing teeth and better oral hygiene. Previous studies on oral health in refugee populations were mostly concerned with children and adolescents. The few examining adults have shown similar trends to our study. In this context, a study in Italy [8] found a significant increase in mean DMFT scores for Moroccan and Yugoslavian refugees with age. The study has also shown a significant increase in mean Oral Hygiene Index (OHI-S) for Yugoslavian refugees with age. Another study in Australia [6] found that the decayed, missing and filled surface (DMFS) index was significantly higher in older and less educated refugees. Females showed significantly higher mean number of filled surfaces (FS) compared to males. The study used the Community Periodontal Index (CPI) to evaluate the periodontal health and oral hygiene. The results showed that older, less educated and male refugees tend to have higher CPI scores. However, the authors were not able to test for statistical significance due to the small numbers of participants in each group.

Among the purposes of this study was to assess the knowledge, attitude and practice of refugees on oral health. The participants had in general a high level of knowledge, however, an emphasis should be put on the importance of tooth flossing as a complimentary method to clean the inter-dental embrasures and the proximal tooth surfaces in addition to the tooth brushing. On average, the participants showed high scores on attitude, still they should be more informed about the established relationship between oral and general health and the importance of regular dental checkups even when they have no pain or acute complaint. On the other hand, participants have generally shown lower scores on practice presenting a gap between their knowledge and practice. This gap could be related to the adversities surrounding the pre- and post-arrival conditions. Nonetheless, it is important for health promotion campaigns to bridge the gap between knowledge and practice and to concentrate more on oral hygiene motivation among refugees in order to establish or re-establish the norms of tooth brushing at least twice a day with preferably fluoride-containing toothpaste.

This study was among the first attempts to tackle the oral health status of refugees in Germany. The study however had certain limitations, among which:

- The use of convenience sampling instead of randomized cluster sampling making the study sample less representative. In addition, the sampling procedure may have led to a selection bias, as study participants recruited in private practices may tend to have a higher occurrence of dental disease in comparison to those recruited in refugee shelters.
- The sensitivity of field oral examinations (for obtaining DMFT scores in particular) without the use of extra diagnostic methods like bitewing radiographs. This may have led to underestimating the prevalence of caries and to increased false negative values especially for caries on the proximal surfaces. In addition, it was only feasible to examine the intra-rater reliability for participants recruited in private practices (a convenience sample). This may have put the resulting intra-class correlation coefficient at risk of bias.
- The social desirability bias with regard to KAP survey, as participants may tend to satisfy the examiner with their answers rather than to express what they believe or practice in their daily life reality.
- The study has examined the association of oral health status with certain socio-demographic characteristics. However, there are other important characteristics that were not analyzed in our study. Some of these could be associated with the population of origin like sugar intake and smoking, others are related to the post arrival adversities like waiting times associated with asylum application and linguistic barriers. These and similar potential associations could be investigated in future studies.

## Conclusions

The present study shows a high prevalence of untreated caries and poor oral hygiene among newly arrived refugees in Germany. It suggests that socio-demographic factors including age, gender and education are associated with the oral health status of the refugees and partly with their knowledge, attitude and practice on oral hygiene. It is important to put emphasis on motivation in oral health promotion campaigns among refugees and to provide them with adequate guidance, preferably in their native language, on how to access oral health care in the host country.

## Abbreviations

AIC: Akaike’s information criterion
BIC: Bayesian information criterion
CPI: Community Periodontal Index
DMFS: Decayed, Missing and Filled Surface Index
DMFT: Decayed, Missing and Filled Teeth Index
DT: Decayed Teeth
FS: Filled Surfaces
FT: Filled Teeth
GLM: Generalized linear model
KAP: Knowledge, Attitude and Practice
MT: Missing Teeth
NBRM: Negative binomial regression model
OHI-S: Oral Hygiene Index
OLR: Ordered logistic regression
RC: Regression Coefficient
SD: Standard Deviation
WHO: World Health Organization
